# Walnut inclusion in a palm oil-based atherogenic diet promotes traits predicting stable atheroma plaque in *Apoe*-deficient mice

**DOI:** 10.3389/fnut.2023.1079407

**Published:** 2023-02-08

**Authors:** Iolanda Lázaro, Joaquim Bobi, Montserrat Cofán, Garyfallia Kapravelou, Antonio J. Amor, Joaquin Surra, Carmen Gómez-Guerrero, Emilio Ortega, Jesus Osada, Ana Paula Dantas, Aleix Sala-Vila

**Affiliations:** ^1^Cardiovascular Risk and Nutrition, Hospital del Mar Medical Research Institute (IMIM), Barcelona, Spain; ^2^Experimental Cardiology, Institut Clínic Cardiovascular, Institut d'Investigacions Biomèdiques August Pi i Sunyer (IDIBAPS), Barcelona, Spain; ^3^Division of Experimental Cardiology, Department of Cardiology, Erasmus MC University Medical Center, Rotterdam, Netherlands; ^4^CIBER de Fisiopatología de la Obesidad y Nutrición, ISCIII, Madrid, Spain; ^5^Translational Research in Diabetes, Lipids and Obesity, IDIBAPS, Barcelona, Spain; ^6^Department of Physiology, Institute of Nutrition and Food Technology (INyTA), Center for Biomedical Research, Center for Research in Sport and Health (IMUDS), Universidad de Granada, Granada, Spain; ^7^Endocrinology and Nutrition Department, Hospital Clínic de Barcelona, Barcelona, Spain; ^8^Department of Producción Animal, Escuela Politécnica Superior de Huesca, Huesca, Spain; ^9^Renal, Vascular and Diabetes Research Lab, IIS-Fundación Jiménez Díaz, Madrid, Spain; ^10^CIBER de Diabetes y Enfermedades Metabólicas, ISCIII, Madrid, Spain; ^11^Department of Bioquímica y Biología Molecular y Celular, Facultad Veterinaria, Instituto de Investigación Sanitaria de Aragón, Universidad de Zaragoza, Zaragoza, Spain

**Keywords:** palm oil, unsaturated fat, nuts, Nrf2, inflammation, efferocytosis

## Abstract

**Introduction:**

The lower rates of cardiovascular disease in Southern Europe could be partially explained by the low prevalence of lipid-rich atheroma plaques. Consumption of certain foods affects the progression and severity of atherosclerosis. We investigated whether the isocaloric inclusion of walnuts within an atherogenic diet prevents phenotypes predicting unstable atheroma plaque in a mouse model of accelerated atherosclerosis.

**Methods:**

Apolipoprotein E-deficient male mice (10-week-old) were randomized to receive a control diet (9.6% of energy as fat, *n* = 14), a palm oil-based high-fat diet (43% of energy as fat, *n* = 15), or an isocaloric diet in which part of palm oil was replaced by walnuts in a dose equivalent to 30 g/day in humans (*n* = 14). All diets contained 0.2% cholesterol.

**Results:**

After 15 weeks of intervention, there were no differences in size and extension in aortic atherosclerosis among groups. Compared to control diet, palm oil-diet induced features predicting unstable atheroma plaque (higher lipid content, necrosis, and calcification), and more advanced lesions (Stary score). Walnut inclusion attenuated these features. Palm oil-based diet also boosted inflammatory aortic storm (increased expression of chemokines, cytokines, inflammasome components, and M1 macrophage phenotype markers) and promoted defective efferocytosis. Such response was not observed in the walnut group. The walnut group’s differential activation of nuclear factor kappa B (NF-κB; downregulated) and Nrf2 (upregulated) in the atherosclerotic lesion could explain these findings.

**Conclusion:**

The isocaloric inclusion of walnuts in an unhealthy high-fat diet promotes traits predicting stable advanced atheroma plaque in mid-life mice. This contributes novel evidence for the benefits of walnuts, even in an unhealthy dietary environment.

## 1. Introduction

Coronary heart disease (CHD) secondary to atherosclerosis is the leading cause of mortality worldwide (1). Mediterranean countries disclose low rates of fatal CHD compared with other countries of Northern Europe (2). In parallel, studies of atheroma plaque composition from different European populations show that an increasing number of lipid-rich, vulnerable coronary plaques, responsible for most culprit lesions, is detected from Southern to Central-Northern Europe (3).

As followed in Mediterranean countries, the Mediterranean diet is characterized by the customarily intake of unsaturated fats. There is consistent epidemiologic evidence from a few small trials conducted nearly 50 years ago that, rather than reducing total dietary fat, replacing saturated fat with unsaturated fats, especially polyunsaturated fat, is effective in reducing CHD through anti-atherosclerotic mechanisms (4). However, given that foods are more than nutrients, the single nutrient approach can be misleading when translating messages into the society (5).

Nuts are an integral part of the Mediterranean diet and of other healthy plant-based diets such as the Dietary Approaches to Stop Hypertension (DASH) diet. In addition to their richness in bioactive phytochemicals (mainly antioxidants and phytosterols), nuts have a favorable fatty acid profile, with an abundant supply of polyunsaturated fatty acids (PUFA), and in the particular case of walnuts, alpha-linolenic acid (C18:3n-3, ALA, the vegetable omega-3 fatty acid) (6), for which there is increasing epidemiologic and experimental evidence of CHD benefits (7, 8). In a randomized controlled crossover trial, short-term (2-week) isocaloric replacement of saturated fat with polyunsaturated fats (from walnuts and vegetable oils) and monounsaturated fats (from vegetable oils) significantly improved atherogenic risk factors (9). In the framework of the PREvención and DIeta MEDiterranea trial (PREDIMED), subjects receiving a Mediterranean diet supplemented with mixed nuts (15 g of walnuts + 7.5 g of almonds + 7.5 g of hazelnuts/day) for 2.4 years showed delayed plaque progression compared to those advised to follow a low-fat (control) diet (10). However, since there was no nutrient substitution in the nut arm, consumption of sources of saturated fat was not reduced compared with the low-fat dietary advice group. Hence, it remains unknown whether sustained isocaloric inclusion of nuts within an unhealthy saturated fat-rich diet prevents the progression of unstable atheroma plaques. Such hypothesis could only be tested by long-term randomized clinical trials, which would be unethical. We addressed this issue in a mouse model of accelerated atherosclerosis in the present work.

## 2. Materials and methods

### 2.1. Dietary intervention and animal model

We used three purified diets formulated and produced in Dr. Jesús Osada’s lab (University of Zaragoza, Spain). A detailed list of ingredients is described in Supplementary Table 1, and nutrient composition is described in Supplementary Table 2. All diets contained 0.2% (w/w) of cholesterol. The first diet (control diet, CD) was standard mouse chow, with 9.6% of the energy supplied as fat, with soybean oil as the sole fat source. The second diet was a high-fat diet (~43% of the total energy supplied as fat) with palm oil as the main fat source (palm oil-based high-fat diet, PO HFD). Finally, the third diet (palm oil + walnuts high-fat diet, PO + W HFD) was also a high-fat diet (~43% of the total energy supplied as fat), but with palm oil partially replaced with walnuts. This diet contained 3% (w/w) of walnuts, which represents a daily dose of 3 g walnuts/kg bodyweight per mouse; considering the higher metabolic rate of mice (11), it would translate into the dose of 30 g/day (one serving) in humans. The walnuts were grounded in a hammer mill until a paste form, and then, carefully mixed with the powdered ingredients by successive premixtures until a homogeneous mass with total powdered ingredients. The diets did not contain any preservatives; therefore, they were stored at −20°C up to their usage.

Male apolipoprotein E-deficient (*Apoe*^−/−^; Jackson Laboratory, Bar Harbor) mice were bred and maintained at the Animal Facility of the Faculty of Medicine of the University of Barcelona. Mice were housed in cages (2–4 animals/cage) in a temperature-controlled room (20–22°C and 12-h light/dark cycle) with environmental enrichment and bedding. Studies were conducted under the 3R principle (replacement, refinement, and reduction) strictly following with the Directive 2010/63/EU of the European Parliament and were approved by the Institutional Animal Care and Use Committee of the University of Barcelona and Generalitat de Catalunya (DAAM 8727). Ten-week-old *Apoe*^−/−^ male mice (*n* = 43) were randomized to receive either CD (*n* = 14), PO HFD (*n* = 15), or PO + W HFD (*n* = 14) diets for 15 weeks (see experimental design, Supplementary Figure 1). We renewed the diets every 3 days to avoid being spoiled. Water was available *ad libitum*. We monitored animals to record body weight (weekly) and food intake (twice a week, a pre-weighed amount of food was placed in the cage feeder, and we weighted remaining food before adding a new pre-weighed amount of food). At the end of the intervention, animals were anesthetized (100 mg/kg ketamine and 10 mg/kg xylazine), euthanized, and saline-perfused. We collected blood samples for biochemistry. We harvested, cleaned, and divided aortas into two parts: the upper aortic root for histological analyses and the thoraco-abdominal fragments for expression analyses.

### 2.2. Biochemistry

Serum total cholesterol, high-density lipoprotein-cholesterol, and triglycerides were measured in CORE Laboratory (Hospital Clínic de Barcelona) by standard enzymatic methods. We assessed the serum ratio cholesteryl ester to free cholesterol by the Cholesterol/Cholesteryl Ester Quantitation Kit (Cat# 428901, Calbiochem, Merck, Darmstadt, Germany), and the serum total antioxidant capacity by the OxiSelect TAC Assay Kit (Cat# STA-360, Cell Biolabs Inc., San Diego CA, United States).

### 2.3. Atherosclerotic lesion analysis

We carefully embedded the upper aortic roots in Tissue-Tek OCT Compound (Sakura Finetek Europe, Alphen aan den Rijn, The Netherlands). We obtained cryosections of aortic roots at 5 μm thickness beginning proximally at the first evidence of the aortic valves at their attachment site of the aorta. To determine the size and extension of atherosclerotic lesions, we stained aortic sections with Oil red O/hematoxylin at 100 μm intervals from 0 to 1,200 μm. We calculated the mean maximal lesion area (μm^2^) and neutral lipid content for each mouse aorta by averaging the values for the three highest consecutive sections. Besides hematoxylin/eosin (H&E) staining, we performed picrosirius red staining to analyze collagen content by measuring bright-field (total collagen) and birefringence to plane-polarized light [type I (orange) and type III (green) collagen]. Regarding immunohistochemical analyses, we detected total macrophages by immunoperoxidase (CD68; Cat# ab53444, Abcam, Cambridge, United Kingdom) and vascular smooth muscle cells (VSMC) by immunofluorescence (α-actin-Cy3; Cat# C6198, Sigma-Aldrich, St. Louis, MO, United States). Finally, calcifications were assessed by von Kossa staining. These parameters are used to determine plaque stability using the Stary score (12), a visual rating of plaque lesions into: grade I (early plaques containing only macrophages); grade II (lesions containing macrophages, VSMCs, and a few cholesterol clefts); grade III (lesions containing macrophages, VSMCs, and numerous cholesterol clefts); grade IV (advanced plaques containing macrophages, VSMCs, and a large necrotic core); or grade V (advanced plaques containing calcified regions).

Next, we determined apoptotic cells in lesions by terminal deoxynucleotidyl transferase dUTP nick end labeling (TUNEL) method by using the *in situ* cell death detection kit (Cat# 12156792910, TMR red, Roche Diagnostics, Rotkreuz, Switzerland) and nuclear counterstain with 4′,6-diamidino-2-phenylindole (DAPI, Sigma-Aldrich). Plaque necrosis was quantified by measuring the blank areas in the intima in picrosirius red-stained sections.

We determined lesional activated nuclear factor kappa B (NF-κB) and nuclear factor (erythroid-derived 2)-like 2 (Nrf2) by *in situ* Southwestern histochemistry using digoxigenin-labeled probes (13, 14).

For colocalization of CCL2 with macrophages and VSMC, double immunofluorescence was performed. Samples were simultaneously incubated with primary antibodies (anti-CCL2; Cat# sc-1,304, Santa Cruz Biotechnology Inc., Santa Cruz, CA; and anti-CD68), followed by secondary antibodies (anti-Goat IgG, AlexaFluor 555, Cat# A32816, Thermo Fisher Scientific; anti-Goat IgG, AlexaFluor 488, Cat# A-11055, Thermo Fisher Scientific; anti-Rat IgG, Alexa Fluor 488, Cat# A-11006, Thermo Fisher Scientific) or anti-αSMA IgG Cy3. For colocalization of LC3B with macrophages and VSMC, double immunofluorescence was performed. Samples were simultaneously incubated with primary antibodies (anti-LC3B, Cat# 2775, Cell Signaling Technology, Beverly, MA, United States; and anti-CD68), followed by secondary antibodies (anti-Rabbit IgG, AlexaFluor 568, Cat# A-11011, Thermo Fisher Scientific; anti-Rabbit IgG, AlexaFluor 488, Cat# 711–545-152, Jackson ImmunoResearch, West Grove, PA, United States) or anti-αSMA IgG Cy3. In both cases, samples were mounted with Fluoromount-G with DAPI (Cat# 00–4959-52, Invitrogen, Thermo Fisher Scientific) and images obtained using a Nikon Eclipse Ni-E Microscope (Nikon, Japan).

Finally, we detected lesional proteolytic-cleavaged form (sol-Mer) of macrophage efferocytosis receptor c-Mer tyrosine kinase (MerTK) by immunohistochemistry with a goat anti-MerTK antibody specific for the ectodomain of mouse MerTK (Cat# AF591, R&D system, Bio-techne, Minneapolis, MN, United States) (15). Concentration used for each antibody in immunohistochemistry can be found in Supplementary Table 3.

Computer-assisted morphometric analyses were performed with Image Pro-Plus (Media Cybernetics, Bethesda, MD) or ImageJ software from the NIH.^1^ The threshold setting for the positive area was equal for all images. In all cases, analyses were conducted in a blinded-manner. We expressed the results as the percentage of positive area versus total area (lipid content, collagen, macrophages, VSMCs, calcification, necrosis, NF-κB, Nrf2, and sol-Mer) or positive nuclei per lesion area (TUNEL).

### 2.4. RNA extraction and real-time PCR analysis

We extracted total RNA from the thoraco-abdominal aorta of mice using the TRI Reagent (Cat# 93289, Sigma-Aldrich, Merck) method and quantified it using a NanoDrop 2000c Spectrophotometer (Thermo Fisher Scientific, Waltham, MA, United States). For each sample, we reversely transcribed 2 μg of total RNA into cDNA using High-Capacity cDNA Reverse Transcription Kit (Cat# 4368814, Applied Biosystems, Foster City, CA, United States). We performed quantitative real-time PCR on a ViiA 7 Real-Time PCR System using Premix Ex Taq Master Mix (Cat# RR39WR, Clontech, Takara, Saint-Germain-en-Laye, France). We used TaqMan probes (Applied Biosystems) and optimized them according to the manufacturer’s protocol (Supplementary Table 4). We determined mouse mRNA levels for NADPH oxidase subunits, and macrophages and VSMCs phenotype markers by amplifying cDNA using PowerUp SYBR Green Master Mix (Cat# A25742, Thermo Fisher Scientific). The sequences of the primers are summarized in Supplementary Table 5. For each sample, we normalized the expression levels of target genes to housekeeping transcripts [*18S rRNA* (TaqMan); *Gapdh* (SYBR)]. We determined the relative expression using the formula 2^-ΔΔCt^, expressing values in arbitrary units.

### 2.5. Protein expression analysis

Aortic tissue proteins were isolated using TRI Reagent (Cat# 93289, Sigma-Aldrich, Merck) according to the manufacturer’s instructions for protein extraction, resolved on SDS-PAGE gels, transferred into polyvinylidene difluoride (PVDF) membranes, and immunoblotted for autophagy-related (ATG)5 (Cat# 12994, Cell Signaling Technology), ATG7 (Cat# 8558, Cell Signaling), beclin-1 (Cat# 3495, Cell Signaling), microtubule-associated protein 1 light chain 3 (LC3B), and sequestosome (SQSTM1)/p62 (Cat# sc-28,359, Santa Cruz Biotechnology Inc.), with β-actin (Cat# A5441, Sigma-Aldrich) as the loading control. We used peroxidase-conjugated secondary antibodies (Jackson ImmunoResearch). Concentration used for each antibody in protein expression analyses can be found in Supplementary Table 6. We quantified blots using Image Lab software (Bio-Rad Laboratories, Hercules, CA, United States). We expressed densitometric values normalized to loading control in arbitrary units or converted to fold-increases versus CD group.

### 2.6. Fatty acids analysis

Samples of diet homogenates (*n* = 5 samples from different batches of each diet) were spiked with the internal standard trinonadecanoin (Merck), and the lipids were extracted with chloroform/methanol (2:1 v/v). Fatty acid methyl esters were prepared as described previously (16). They were separated by gas chromatography with an Agilent 7890 Gas Chromatograph HP 6890 equipped with a 30 m × 0.25 μm × 0.25 mm SupraWAX-280 capillary column (Teknokroma, Barcelona, Spain), an autosampler, and flame ionization detection. We expressed fatty acids both as the percentage of the total amount of fatty acids and as a concentration (mg fatty acid/g chow).

### 2.7. Statistics

We calculated the study sample size based on previous results from the study conducted by Nergiz-Ünal and collaborators (17). A sample of 12 mice per group achieved 80% power to detect differences using a Student’s *t*-test for two independent samples with a significance level of 5%, and assuming that a size of the aortic lesion of 200 units (control group) and 80 units (test group), and a standard deviation of both groups of 15 units. Considering a 20% loss, we needed 14 mice per group.

We expressed values as mean ± SEM or as median and interquartile range. We used the Kolmogorov–Smirnov test to assess normal distribution. We log-transformed skewed variables for further parametric analyses. We explored the effect of the intervention on weekly weight gain by ANOVA, using a general linear model (GLM) with repeated-measures and Greenhouse–Geisser correction of *p* values. We compared groups with one-way ANOVA with Bonferroni *post hoc* test for other variables. We used a linear model to assess the association between fasting non-HDL-c and plaque lipid content, calculating the standardized regression coefficients (β) and the *p* values. In all cases, *p* values were two-sided, and we considered differences statistically significant if *p* < 0.05. Analyses were performed using SPSS software, release 19.0 (IBM Corp., Armonk, NY, United States). Figures were built using R Software (R Foundation for Statistical Computing; http://www.r-project.org/).

## 3. Results

All animals completed the intervention. Feed intake (Supplementary Figure 2A) was significantly lower in PO + W HFD (2.75 ± 0.03 g per day, in average) than in the CD (3.15 ± 0.02; *p* < 0.001) and PO HFD (3.04 ± 0.02; *p* < 0.001). However, we found no significant differences for body weight change during the intervention when considering repeated measurements (*p* = 0.204; Supplementary Figure 2B).

### 3.1. The inclusion of walnuts partially reverts phenotypes predicting unstable plaque induced by palm-oil diet

**Figure 1:**
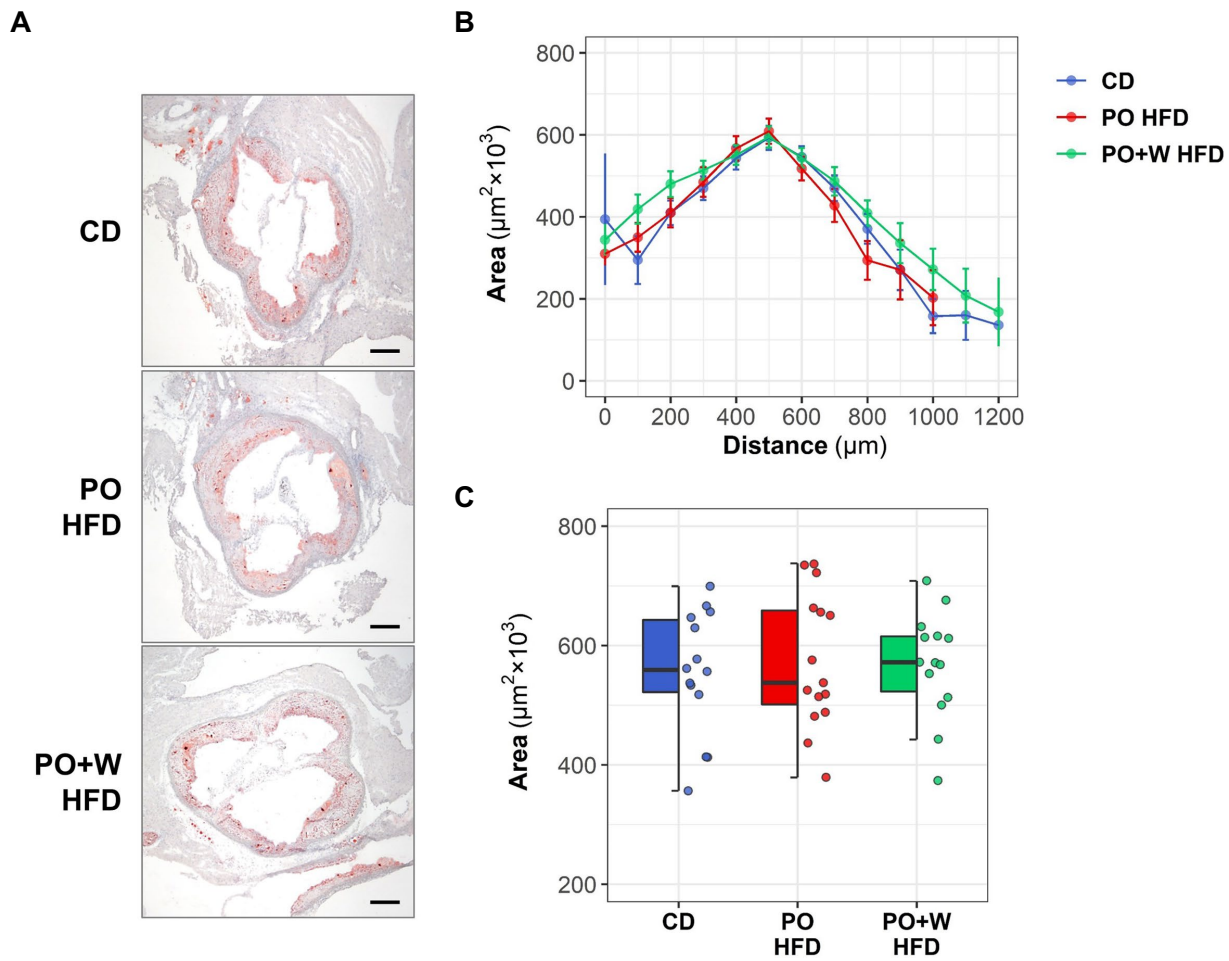
Effect of three different diets on atherosclerotic plaque extension and size. *Apoe*^−/−^ mice received control diet (CD), palm oil-based high-fat diet (PO HFD), or isocaloric palm oil + walnuts high-fat diet (PO + W HFD) diet for 15 weeks. **(A)** Representative images (magnification x40; scale bars, 200 μm) of Oil red O/hematoxylin staining in aortic root sections. **(B)** Quantification of the extent of atherosclerotic lesions within the aorta (11–13 sections per mice); the line-plot shows results as mean ± SEM. **(C)** Mean maximal lesion area in each group of intervention (2–3 sections per mice); dot and box plot shows individual values and depicts the median (horizontal bar), interquartile range (IQR, hinges) and 1.5 × IQR (whiskers). CD, control diet (*n* = 14); PO HFD, palm oil-based high-fat diet (*n* = 15); and PO + W HFD, palm oil + walnuts high-fat diet (isocaloric inclusion of 3% of walnuts at expense of palm oil, *n* = 14).

**Figure 2:**
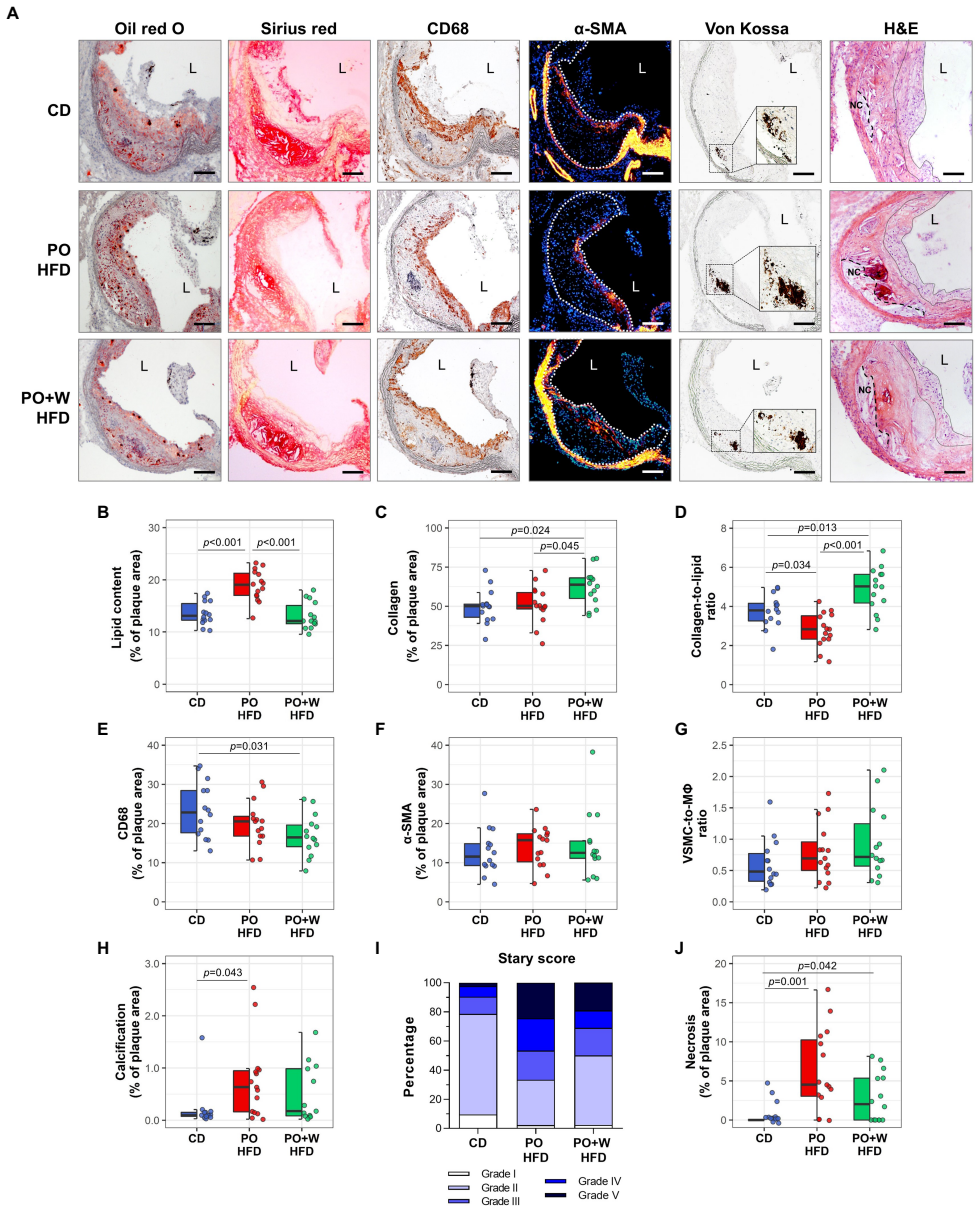
Inclusion of walnuts contributes to prevent phenotypes predicting plaque instability and progression induced by high-fat diet. **(A)** Representative images of Oil red O/hematoxylin, Sirius red, CD68, α-SMA, Von Kossa, and hematoxylin–eosin (H&E)-stained sections from each group of intervention [magnification × 100; scale bars, 100 μm; high-magnification fields (squared areas)]. **(B–H)** Quantification of parameters used to rate plaque stability, including intraplaque lipid (Oil red O, **B**), collagen (Sirius red, **C**), collagen-to-lipid ratio **(D)**, macrophages (CD68, **E**), VSMC (α-SMA, **F**), VSMC-to-macrophage (MΦ) ratio **(G)**, and calcification (Von Kossa, **H**). **(I)** Classification of aortic lesions according to the Stary method (grades I–V). **(J)** Measurement of intraplaque necrotic area in Sirius red-stained histological sections. Dot and box plots show individual values and depict the median (horizontal bar), interquartile range (IQR, hinges) and 1.5 × IQR (whiskers). CD, control diet (*n* = 14); PO HFD, palm oil-based high-fat diet (*n* = 15); PO + W HFD, palm oil + walnuts high-fat diet (isocaloric inclusion of 3% of walnuts at expense of palm oil, *n* = 14). *p* values obtained by one-way ANOVA (after log-transformation) with Bonferroni *post hoc* correction. Dashed lines in α-SMA images define atherosclerotic lesions. Dashed and dotted lines in H&E images delimit necrotic cores (NC) and fibrous cap regions, respectively. L, lumen.

Regarding the quantitative assessment of aortic root sections stained with Oil red O/hematoxylin (Figure 1A), we observed no statistically significant differences among groups for lesion extension along the aorta (Figure 1B) or mean maximal lesion area (in μm^2^ × 10^3^: CD, 555 ± 28; PO HFD, 575 ± 29; PO + W HFD: 568 ± 24; *p* = 0.869; Figure 1C). There was, however, a protective effect of the walnut diet on several histology-assessed predictors of plaque stability, namely the content of lipid, collagen, macrophages, VSMC, and calcified areas (Figure 2A). As shown in Figure 2B, atherosclerotic lipid deposition in PO HFD (% positive area versus total area, 19.05 ± 0.79) was significantly higher than in CD (13.53 ± 0.60), while walnut inclusion prevented such increase (13.13 ± 0.70). Picrosirius red staining showed that plaques from PO + W HFD mice had a significantly higher presence of total collagen when compared to the other groups (Figure 2C). This overall translated into a higher collagen-to-lipid ratio in the PO + W HFD group (Figure 2D). Further analysis of collagen under polarized light microscopy (Supplementary Figure 3A) revealed a higher proportion of type I (mature, thicker orange-red fibers) to type III (immature, thinner yellow-green fibers) collagen in PO + W HFD compared with CD and PO HFD (Supplementary Figure 3B). Atherosclerotic lesions of mice receiving walnuts also showed lower macrophage content (Figure 2E). No significant among-group differences were observed for neointimal VSMCs content (Figure 2F), and the contractile phenotype VSMCs (by studying mRNA expression of contractile (calponin) marker, Supplementary Figure 4), or the VSMC-to-macrophage ratio (Figure 2G). However, differences were observed between PO HFD and PO + W HFD for the mRNA expression of secretory marker of VSMCs (osteopontin; Supplementary Figure 4). Finally, Von Kossa staining revealed the presence of calcified deposits in the neointima of atherosclerotic lesions of PO HFD and PO + W HFD mice, being significantly higher in PO HFD than CD (Figure 2H). The five parameters (lipid, collagen, macrophages, VSMCs, and calcification) are used for the visual rating of the Stary score (12). As depicted in Figure 2I, dietary intervention influenced the proportion of early lesions (grades I and II) to advanced plaques (grades IV and V) in *Apoe*^−/−^ mice. After 15 weeks of intervention, 79% of lesions in the CD group were rated as early ones, while advanced plaques accounted for 10% of the total. Such proportion was inverted in PO HFD, with 33% being early lesions and 47% advanced ones. The inclusion of walnuts increased the proportion of early lesions (50%) at the expense of advanced ones (31%).

Finally, given that defective clearance of apoptotic cells can lead to plaque necrosis and promote plaque disruption in advanced atherosclerotic lesions, we assessed apoptosis and necrosis. No statistically significant differences were observed among groups for TUNEL-positive cells (*p* = 0.645; Supplementary Figure 5). In contrast, both high-fat diets induced a significantly higher proportion of necrotic areas in the lesion compared to the control diet. However, no statistically significant differences were observed between the two high-fat diets (Figure 2J).

### 3.2. The effect of walnuts on plaque lipid content is beyond changes in non-HDL-c

**Figure 3:**
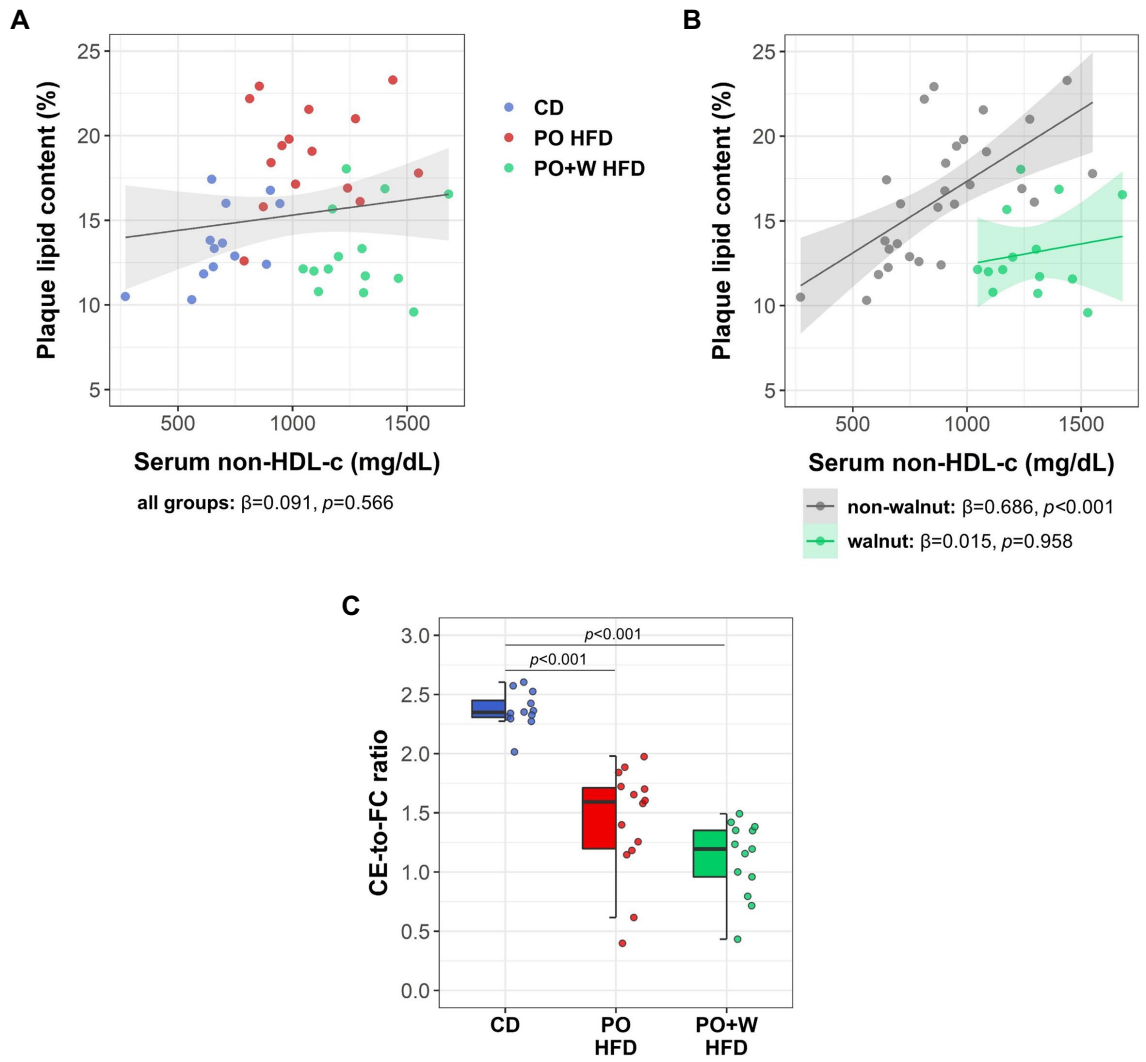
Effect of adding walnuts in the associations between serum non-HDL-c and intraplaque lipid. Scatterplots depicting the associations between serum non-HDL-cholesterol (non-HDL-c) and plaque lipid content considering all the mice together **(A)** and after grouping all mice into either “non-walnut intervention” (control diet and palm oil high-fat diet grouped together; grey) or “walnut intervention” (palm oil + walnuts high-fat diet, green) **(B)**. Lines indicate the regression line and the 95% confidence intervals for each association. The standardized regression coefficients (β) and the *p* values were computed using a linear model. **(C)** Ratio of serum cholesteryl ester (CE) to free cholesterol (FC). Dot and box plots show individual values and depict the median (horizontal bar), interquartile range (IQR, hinges) and 1.5 × IQR (whiskers). CD, control diet (*n* = 14); PO HFD, palm oil-based high-fat diet (*n* = 15); PO + W HFD, palm oil + walnuts high-fat diet (isocaloric inclusion of 3% of walnuts at expense of palm oil, *n* = 14). *p* values obtained by one-way ANOVA (after log-transformation) with Bonferroni *post hoc* correction.

Because of the long-known lipid-lowering effect of nut consumption, we searched for differences on serum non-HDL-c. Mice on control diet showed significantly lower non-HDL-c compared to those from other groups (in mg/dl: CD, 688 ± 124; PO HFD, 1077 ± 229; PO + W HFD, 1270 ± 147; CD vs. PO HFD and PO + W HFD; *p* < 0.001, both), whereas no differences were found for non-HDL-c between the two groups of high-fat diet. We next plotted serum non-HDL-c against plaque lipid content. The inclusion of all groups revealed no significant association (Figure 3A). However, when grouping mice into either “non-walnut intervention” (CD and PO HDF) or “walnut intervention” (PO + W HDF), a significant direct association was found only for the first group (Figure 3B). This finding is suggestive that the effects of walnuts in plaque lipid content might be beyond changes in circulating non-HDL-c. Finally, both high-fat diets reduced the ratio cholesteryl ester to free cholesterol compared with the control diet (*p* < 0.001, both), without statistically significant differences between high-fat groups (Figure 3C).

### 3.3. The inclusion of walnuts prevents plaque inflammation and defective efferocytosis, but not oxidative stress or autophagy

**Figure 4:**
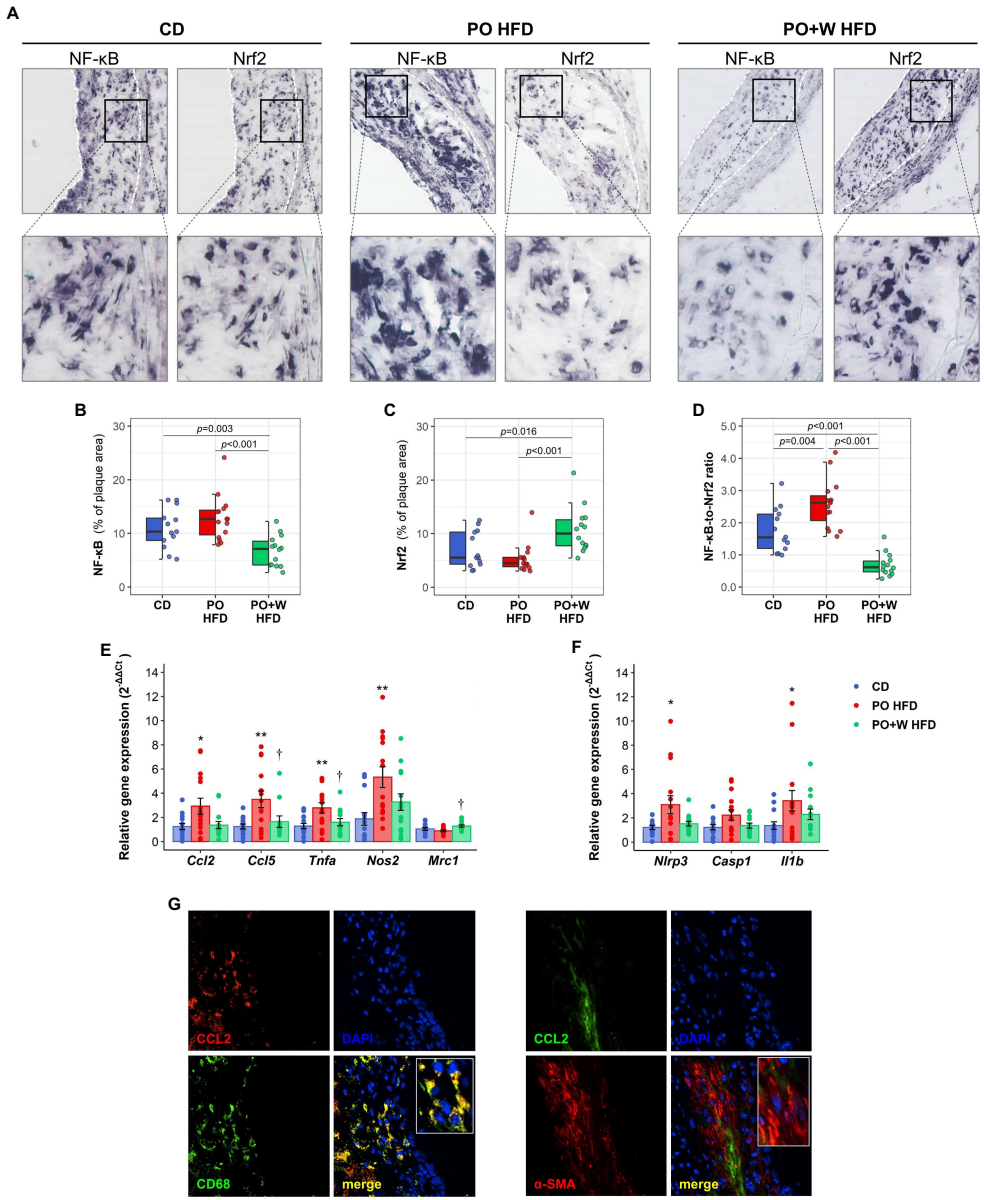
Inclusion of walnuts attenuates inflammation in atherosclerotic lesions. **(A)** Representative images and high-magnification fields of *in situ* detection of activated NF-κB and Nrf2 by Southwestern histochemistry in aortic sections. Dashed white lines delimit atherosclerotic lesion areas. Quantitative analysis of percentage of NF-κB **(B)** and Nrf2 **(C)** lesional activation, and individual NF-κB-to-Nrf2 ratio **(D)**. Quantitative real-time PCR analysis of chemokines, cytokines, M1 and M2 markers **(E)** and inflammasome components **(F)** in mouse aorta. Data normalized by 18S rRNA are expressed as relative gene expression (using 2^–ΔΔCt^ method). Dot and box plots show individual values and depict the median (horizontal bar), interquartile range (IQR, hinges), and 1.5 × IQR (whiskers). Results in bar plots are presented as individual data points and mean ± SEM of the intervention groups. **(G)** Representative images and high-magnification fields (rectangular areas) in atherosclerotic lesion sections showing immunodetection of CD68+ macrophages (left panel) and α-SMA+ VSMC (right panel), and colocalization with CCL2. CD, control diet (*n* = 14); PO HFD, palm oil-based high-fat diet (*n* = 15); PO + W HFD, palm oil + walnuts high-fat diet (isocaloric inclusion of 3% of walnuts at expense of palm oil, *n* = 14). L, lumen. *p* values obtained by one-way ANOVA (after log-transformation) with Bonferroni *post hoc* correction. ^*^p < 0.05, and ^**^p < 0.01 vs. CD, ^†^*p* < 0.05 vs. PO HFD.

Nuclear factor kappa B (NF-κB) controls inflammation (a major trigger of atheroma plaque progression and/or disruption) by regulating gene expression of inflammatory mediators (such as chemokines and cytokines). As a result, many drugs to treat atherosclerosis target NF-κB (18). Nrf2 is an upstream regulator that not only controls the oxidative stress response (19), but also suppresses inflammation by regulating cytokine production and crosstalk with the NF-κB signaling pathway (20). We investigated by *in situ* Southwestern histochemistry (13, 14) whether experimental diets induced a different activation in atherosclerotic lesions of these two key transcription factors (Figure 4A). Atherosclerotic lesions from PO + W HFD mice showed a significant reduction of NF-κB-positive nuclei compared with other groups (decrease 32 and 48% compared with CD and PO HFD, respectively; Figure 4B). Nrf2 activation showed opposite results (Figure 4C) with 1.5- and 2.0-fold increases in PO + W HFD compared with CD and PO HFD, respectively, thus translating into a significantly lower NF-κB-to-Nrf2 ratio (Figure 4D).

We next explored the downstream effect of activation or inhibition of these transcription factors, by assessing gene expression in aortic tissue by real-time PCR. In line with NF-κB activation, mice fed with PO HFD showed a statistically significant upregulation of pro-inflammatory genes including chemokines (*Ccl2* and *Ccl5*), cytokines (*Tnfa*), and M1 macrophage phenotype marker (*Nos2*) when compared with those receiving control diet (Figure 4E). The inclusion of walnuts partially prevented such increases, in particular regarding *Ccl5* and *Tnfa*. No statistically significant differences were observed between CD and PO + W HFD for any gene. Mice from PO + W HFD also showed a higher expression of anti-inflammatory markers and a pro-resolving M2 phenotype (*Mrc1*) when compared to PO HFD mice (Figure 4E). In line with this, mice from PO HFD, but not those from PO + W HFD, showed a significant increase in the gene expression of two inflammasome components (*Nlrp3, Il1b*; Figure 4F). Finally, double immunofluorescence in atherosclerotic lesion sections revealed CCL2 protein expression colocalizing with CD68+ macrophages (Figure 4G, left panel), rather than with α-SMA+ VSMC-stained areas (Figure 4G, right panel).

Despite the differential Nrf2 activation by PO + W HFD, we observed no significant changes in gene expression of proteins involved in the production of reactive oxygen species [NADPH oxidase (NOX) subunits: *Nox1*, *Nox4*, and *p22*], or antioxidant defense enzymes (*Sod1*, *Cat*, and *Hmox1*; Supplementary Figure 6A). In line with the absence of changes in most modulators of redox status, we found no differences among groups in serum total antioxidant capacity (Supplementary Figure 6B).

We then focused on autophagy, a key player in controlling the onset and progression of atheroma plaque (21), the status of which is also modulated by Nrf2 activation (14). To investigate the putative involvement of autophagy in walnuts-mediated atheroprotection, we analyzed by Western blot the aortic expression of proteins involved in critical steps of autophagy, from initiation (Beclin1) to autophagosome formation (ATG5, ATG7, and LC3B) and targeting polyubiquitinated proteins for degradation (SQSTM1). As shown in Supplementary Figures 7A,B, no statistically significant differences were observed among groups. Finally, double immunofluorescence in atherosclerotic lesion sections revealed LC3B protein expression colocalizing with CD68+ macrophages (Supplementary Figure 7C, left panel), rather than with α-SMA+ VSMC-stained areas (Supplementary Figure 7C, right panel).

**Figure 5:**
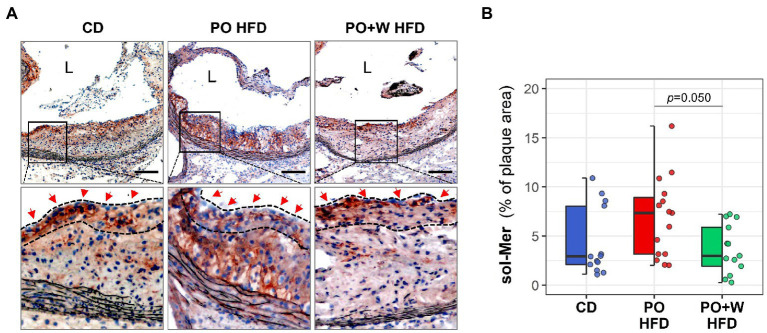
Inclusion of walnuts attenuates defective efferocytosis in atherosclerotic lesions. **(A)** Representative images [magnification × 100; scale bars, 100 μm; and high-magnification fields (squared areas)] and **(B)** Quantitative analysis of histochemical detection of sol-Mer in aortic sections. Dashed lines and red arrows in high-magnification fields define fibrous cap regions. Dot and box plots show individual values and depict the median (horizontal bar), interquartile range (IQR, hinges) and 1.5 × IQR (whiskers). Results in bar plots are presented as individual data points and mean ± SEM of the intervention groups. CD, control diet (*n* = 14); PO HFD, palm oil-based high-fat diet (*n* = 15); PO + W HFD, palm oil + walnuts high-fat diet (isocaloric inclusion of 3% of walnuts at expense of palm oil, *n* = 14). *p* values obtained by one-way ANOVA (after log-transformation) with Bonferroni *post hoc* correction. L, lumen.

The resolution of acute inflammation involves attenuating the production of pro-inflammatory mediators and enhancing processes such as efferocytosis, mediated by macrophage receptors such as MerTK. Therefore, we assessed the levels of inactive sol-Mer, as a marker of MerTK cleavage and defective efferocytosis (15, 22). Of note, we noticed a different distribution of sol-Mer protein in atherosclerotic lesions. Whereas sol-Mer was detected throughout the lesion in PO HFD plaques, it was mainly found in the fibrous cap region in CD and PO + W HFD ones (high-magnification fields inserts, Figure 5A). In addition, we observed that atherosclerotic lesions from the PO + W HFD group showed a significant decrease of sol-Mer compared with lesions of PO HFD mice (Figure 5B), mirroring improved efferocytosis.

## 4. Discussion

In this study, we explored whether the isocaloric replacement of palm oil (a source of saturated fat) with walnuts (a source of polyunsaturated fat besides other salutary bioactives) affects the progression of experimental atherosclerosis. Compared to the palm oil-based diet, the inclusion of walnuts in the high-fat diet for 15 weeks did not modify either the size or the extension of atherosclerotic lesions. However, it translated into lower intraplaque lipid content and macrophage infiltration, less calcification, and increased collagen content. This overall resulted in a phenotype predicting a more stable plaque. When further exploring for mechanistic insights, we observed that the inclusion of walnuts to PO HFD attenuated inflammatory response and defective efferocytosis, while oxidative stress and autophagy were essentially unaffected. The role of walnuts in changes observed upon walnut inclusion into a palm oil-based high-fat diet might be either active (supply of salutary bioactives) or passive (given its satiating effect, they promote consumption of less food). All in all, favoring phenotypes that predict stability of advanced atheroma plaques (even in a background of unhealthy dietary fat) adds to the long-known vasculoprotective properties of walnuts (Supplementary Figure 8).

The importance of our findings relies on three aspects. First, it supports that, in terms of atheroma plaque phenotype, dietary fat quality prevails over the quantity. We replaced sources of saturated fat with an amount of walnuts equivalent to 30 g/day in humans, the amount of nuts that delayed atherosclerosis progression in a sub-cohort of the PREDIMED trial (10). In this regard, walnuts provide unsaturated fats (including omega-3) besides other non-lipidic bioactives that also contribute to the cardiovascular protection (23). Second, ours is an advanced yet pre-clinical model of atherosclerosis. We observed that diet significantly affects plaque phenotype in 25-week-old mice, which can be considered mid-life adults. This reinforces the idea that healthy dietary habits should be implemented as soon as possible in life to significantly reduce the prevalence of vulnerable atheroma plaques at mid-life, the harbinger of future atherothrombotic cardiovascular events. Finally, the use of an animal model (our study would be unethical in humans) enabled us to search for novel mechanistic insights.

Although other studies have described the influences of dietary fat content in atherosclerosis, they mainly focused on changes in circulating lipids and plaque progression. Surra et al. reported the benefits of a 12-week diet containing mixed nuts (almonds, hazelnuts, and walnuts in a proportion of 1:1:2, respectively) in preventing atherosclerosis (only in females) and improving blood lipids (in both sexes) in comparison with an isocaloric palm oil-based high-fat diet (11). Similarly, male mice receiving a diet supplemented with walnuts for 8 weeks showed benefits on blood lipids, atherosclerotic lesion size, and aortic expression of macrophage scavenger receptor compared with those receiving a sunflower-rich isocaloric diet (17). Of note, in the latter, such changes were not observed in mice receiving a diet supplemented with walnut oil, suggesting that non-lipidic phytochemicals from walnuts might be the major drivers of the observed effect. A key mechanism for the effects attributed to these non-lipidic phytochemicals is the ability to induce of the redox-sensitive transcription factor Nrf2 (24). Growing evidence on the interplay between nuts and Nrf2 (25) led us to determine whether adding walnuts to a PO HFD would target Nrf2 and lead to a concerted upregulation of cytoprotective pathways in the vasculature. This approach has already been tested for pharmacological agents, found able to mitigate atherosclerosis progression (14). As expected, we found an augmented activation of Nrf2 in the experimental diet containing walnuts. However, such an increase did not translate into changes in oxidative stress and autophagy. In contrast, we observed a significant anti-inflammatory effect of walnuts when added to the PO HFD. Nrf2 is known to contribute to modulate inflammation both on its own and through its functional crosstalk with NF-κB pathway (26). We observed that adding walnuts to the PO HFD induced a differential lesional activation of NF-κB (downregulation) and Nrf2 (upregulation). We speculate that the effect of walnuts on inflammation stems from the impact on these two cellular pathways.

In this sense, our results are concurrent with those suggesting that targeting chemokines, cytokines, and NLRP3 inflammasome activation might be instrumental in reducing atherothrombotic cardiovascular events (27). The Canakinumab Anti-inflammatory Thrombosis Outcomes Study (CANTOS) trial showed that the pharmacological blockage of IL-1β protected from recurrent atherothrombotic cardiovascular events in patients with a history of myocardial infarction, independently of its lipid lowering-effects (28, 29). Similar results were observed for oral-administered colchicine (30), a drug that affects NLRP3 inflammasome activation. When it comes to nuts, a significant reduction of circulating IL-1β has been reported after the dietary supplementation with mixed nuts (compared to the advised low-fat diet) (31) or walnuts (compared to walnut-free diet) (32). The main novelty of our approach (unethical in humans) is that dietary inclusion of walnuts within an unhealthy diet promoting NLRP3 inflammasome activation (33) tackles such activation, hence attenuating the production of pro-inflammatory mediators.

In addition, the inclusion of walnuts also maintained efferocytosis, which is the ability of macrophages to quickly remove apoptotic and necrotic cells. Although preventing defective efferocytosis is a promising therapeutic strategy for atherosclerosis, clinical research is still at the initial stage (34). In overall, the concerted attenuation of production of pro-inflammatory mediators, the prevention of defective efferocytosis, and the promotion of pro-resolving M2 phenotype seems to favor the resolution of acute inflammation in the mice receiving walnuts, translating into a phenotype predicting less unstable atheroma plaque.

Despite the novelties and relevance of our data, we are aware of the limitations of our study. Firstly, we only tested experimental diets in male mice to exclude the differences in the molecular pathways involved in suppressing and stimulating plaque development between sexes (35). We recognize that the effect of the diet may have been different in female mice and endorse the idea of designing new protocols to determine possible sex-associated differences in the role of a walnut-rich diet in the progression of atherosclerotic plaque. Secondly, there are other murine models of accelerated atherosclerosis. We selected *Apoe^−/−^* mice because we considered that the absence of ApoE exacerbates the inflammatory environment caused by a high-fat diet in mice (36). Finally, we tested whether the inclusion of walnuts within an unhealthy diet prevented the onset of traits predicting unstable plaque, rather than plaque regression.

In conclusion, we found that in a preclinical model of accelerated atherosclerosis, the inclusion of walnuts into a palm oil-based high fat diet without changing the total amount of dietary fat resulted in a phenotype predicting a more stable advanced atheroma plaque. This overall provides novel evidence on the mechanisms by which regular walnut consumption might protect against atherothrombotic cardiovascular diseases, even in an unhealthy background.

## Data availability statement

The original contributions presented in the study are included in the article/Supplementary material, further inquiries can be directed to the corresponding authors.

## Ethics statement

The animal study was reviewed and approved by Institutional Animal Care and Use Committee of the University of Barcelona Generalitat de Catalunya (DAAM 8727).

## Author contributions

IL: conceptualization, methodology, investigation, data curation, formal analysis, and writing–original draft. JB, MC, GK, and AA: investigation, methodology, and writing–review and editing. JS and JO: methodology, resources, and writing–review and editing. CG-G: methodology, investigation, and writing–review and editing. EO: conceptualization, resources, and writing–review and editing. AD: investigation, methodology, resources, and writing–review and editing. AS-V: conceptualization, methodology, investigation, data curation, formal analysis, project administration, funding acquisition, and writing–original draft. All authors contributed to the article and approved the submitted version.

## Funding

AS-V was supported by the CWC and the Instituto de Salud Carlos III (ISCIII-FIS-FEDER grant PI15/01014). CG-G is supported by the Spanish Ministry of Science and Innovation (PID2021-127741OB-I00). The funding agencies had no involvement in the study design, data collection, analyses, interpretation of the data, or writing of the manuscript.

## Conflict of interest

Research supported by the California Walnut Commission (CWC, Folsom, CA), which had no involvement in the study design, data collection, analyses, interpretation of the data or writing of the manuscript. AS-V has received research funding through his institution and support to attend professional meetings from the California Walnut Commission (CWC, Folsom, CA).

The remaining authors declare that the research was conducted in the absence of any commercial or financial relationships that could be construed as a potential conflict of interest.

## Publisher’s note

All claims expressed in this article are solely those of the authors and do not necessarily represent those of their affiliated organizations, or those of the publisher, the editors and the reviewers. Any product that may be evaluated in this article, or claim that may be made by its manufacturer, is not guaranteed or endorsed by the publisher.

## Footnotes

^1^ http://rsbweb.nih.gov/ij/
