# Supplementary material for: Walnut inclusion in a palm oil-based atherogenic diet promotes traits predicting stable atheroma plaque in Apoe-deficient mice
Source: Front Nutr. 2023 Feb 8;10:1079407. doi: 10.3389/fnut.2023.1079407 (PMC9944036; doi:10.3389/fnut.2023.1079407)
Supplement: Supplementary file 1 [file Data_Sheet_1.pdf]

Supplementary Material

Supplementary Table 1. Composition of purified diets used in the study

Ingredient	**Control diet (CD)**		**Palm oil-based high-fat diet (PO HFD)**		**Palm oil + walnuts high-fat diet (PO+W HFD)**	
	g/kg diet	% total kcal	g/kg diet	% total kcal	g/kg diet	% total kcal
Corn starch	466.8	48.3	371.6	30.1	367.9	30.1
Maltodextrin	154.7	16.1	123.7	10.0	122.5	10.0
Casein	139.7	15.6	111.7	9.7	110.6	9.7
Cellulose	49.9	0.0	39.9	0.0	39.5	0.0
Sucrose	99.8	10.4	79.8	6.5	79.0	6.5
Mineral Mix	34.9	0.0	27.9	0.0	27.7	0.0
Vitamin Mix	10.0	0.0	8.0	0.0	7.9	0.0
Choline bitartrate	2.5	0.0	2.0	0.0	2.0	0.0
L-cysteine	1.8	0.0	1.4	0.0	1.4	0.0
Cholesterol	2.0	0.0	2.0	0.0	2.0	0.0
Soybean oil	39.9	9.7	31.9	6.0	21.7	4.1
Refined palm oil	0.0	0.0	200.0	37.7	188.1	35.7
Ground peeled walnut	0.0	0.0	0.0	0.0	29.7	3.9

Supplementary Table 2. Breakdown of energy and fat of the three experimental diets

Nutrient	**Control diet (CD)**		**Palm oil-based high-fat diet (PO HFD)**		**Palm oil + walnuts high-fat diet (PO+W HFD)**	
Total energy, kcal/kg	3849.09		4937.73		4897.75	
Fatty sources of energy, g/kg	39.92		231.92		229.13[a]	
Energy from fat, %	9.47		43.68		43.51	
Fatty acid breakdown[b]	% total FA	mg FA/g chow	% total FA	mg FA/g chow	% total FA	mg FA/g chow
SFA	18.4 ± 0.2	6.7 ± 0.1	47.4 ± 0.1	94.7 ± 3.2	45.2 ± 0.5	83.8 ± 2.1
MUFA	26.4 ± 0.1	9.6 ± 0.1	37.2 ± 0.2	74.1 ± 2.4	35.8 ± 0.5	66.7 ± 2.2
PUFA	55.2 ± 0.2	20.2 ± 0.3	15.4 ± 0.0	31.1 ± 1.1	19.0 ± 0.4	36.0 ± 1.9
C18:2n-6	50.1 ± 0.1	18.3 ± 0.3	14.5 ± 0.0	29.0 ± 1.0	17.1 ± 0.3	32.0 ± 1.7
C18:3n-3	5.0 ± 0.1	1.8 ± 0.0	0.9 ± 0.0	1.7 ± 0.1	1.8 ± 0.3	3.5 ± 0.2

SFA, saturated fatty acids; MUFA, monounsaturated fatty acids; PUFA, polyunsaturated fatty acids.
[a]Calculation made considering that 100 g of walnut supply 65.2 g of fat. Source: https://fdc.nal.usda.gov/fdc-app.html#/food-details/170187/nutrients, last accessed December 9th 2021.
[b]Assessed by gas-chromatography (n=5 samples from different batches of each diet), as described in "Materials and Methods" section.

Supplementary Table 3. Antibodies used in immunohistochemical analysis

Primary antibody			Application	Secondary antibody		
**Antigen**	**Brand (reference)**	**Dilution**		**Antigen IgG**	**Brand (reference)**	**Dilution**
α-actin-Cy3	Sigma-Aldrich (Cat# C6198)	1/200	IF	---	---	
CD68	Abcam (Cat# ab53444)	1/500	IHC	Anti-Rat IgG, Biotin	Jackson ImmunoResearch (Cat# 712-065-150)	1/800
		1/50	IF	Anti-Rat IgG, Alexa Fluor 488	Thermo Fisher Scientific (Cat# A-11006)	1/200
CCL2/MCP-1	Santa Cruz Biotechnology (Cat# sc-1304)	1/30	IF	Anti-Goat IgG, AlexaFluor 555	Thermo Fisher Scientific (Cat# A32816)	1/200
				Anti-Goat IgG, AlexaFluor 488	Thermo Fisher Scientific (Cat# A-11055)	1/200
LC3B	Cell Signaling Technology (Cat# 2775)	1/100	IF	Anti-Rabbit IgG, AlexaFluor 568	Thermo Fisher Scientific (Cat# A-11011)	1/200
				Anti-Rabbit IgG, AlexaFluor 488	Jackson ImmunoResearch (Cat# 711-545-152)	1/200
MerTK	R&D system (Cat# AF591)	1/50	IHC	Anti-Goat IgG, Biotin	Jackson ImmunoResearch (Cat# 805-065-180)	1/400

IF, immunofluorescence; IHC, immunohistochemistry.

Supplementary Table 4. TaqMan gene expression assays

Gene symbol	Gene name	Accession number	Assay ID	Amplicon length	Junction
Ccl2	chemokine (C-C motif) ligand 2	NM_011333.3	Mm00441242_m1	74	Exons 1-2
Ccl5	chemokine (C-C motif) ligand 5	NM_013653.3	Mm01302428_m1	71	Exons 2-3
Tnfa	tumor necrosis factor alpha	NM_001278601.1	Mm00443258_m1	81	Exons 1-2
Nlrp3	NLR family, pyrin domain containing 3	NM_145827.3	Mm00840904_m1	84	Exons 2-3
Casp1	caspase 1	NM_009807.2	Mm00438023_m1	99	Exons 3-4
Il1b	interleukin 1 beta	NM_008361.3	Mm00434228_m1	90	Exons 3-4
Sod1	superoxide dismutase 1, soluble	NM_011434.1	Mm01344233_g1	71	Exons 4-5
Cat	catalase	NM_009804.2	Mm00437992_m1	64	Exons 8-9
Hmox1	heme oxygenase 1	NM_010442.2	Mm00516005_m1	69	Exons 2-3
18S	eukaryotic 18S rRNA	X03205.1	4310893E	187	----

Supplementary Table 5. Gene expression assays for SYBR

Gene symbol	Gene name	Accession number	Sequence	Amplicon length	Junction	[Primer]	Efficiency
Nox1	NADPH oxidase 1	NM_172203.2	For: TTTCTCTCCCGAAGGACCTCT<br>Rev: AGCCTCCCTAGGAGCAATCT	52	Exons 1-2	1 µM	84
Nox2	cytochrome b-245, beta polypeptide	NM_007807.5	For: GGGAACTGGGCTGTGAATGA<br>Rev: CTGGCAGCAGGATCAGCATA	208	Exons 1-3	1 µM	92
Nox4	NADPH oxidase 4	NM_001285833.1	For: CCCTGGCACAGCAGAGTATG<br>Rev: ATCCCGGAATCGTTCTGTCC	171	Exons 12-15	1 µM	107
p22phox	cytochrome b-245, alpha polypeptide	NM_001301284.1	For: ATGGAGCGATGGTTGTCGG<br>Rev: AATGGGAGTCCACTGCTCAC	126	Exons 3-5	1 µM	89
Nos2	nitric oxide synthase 2, inducible	NM_001313921.1	For: GGAGCGCTCTAGTGAAGCAA<br>Rev: CCTCACATACTGTGGACGGG	152	Exons 2-3	1 µM	90
Mrc1	mannose receptor, C type 1	NM_008625.2	For: GTGGAGTGATGGAACCCCAG<br>Rev: CTGTCCGCCCAGTATCCATC	120	Exons 8-9	1 µM	95
Ost	osteopontin	NM_001204201.1	For: ATCCTTGCTTGGGTTTGCAG<br>Rev: ACTGCCAATCTCATGGTCGT	138	Exons 1-2	1 µM	114
Calp	calponin 1	NM_009922.4	For: CGGTGTGCACTGGTTCTACT<br>Rev: GCTACTCAACTGCTCTGCCA	63	Exon 7	1 µM	98
Gapdh	glyceraldehyde-3-phosphate dehydrogenase	NM_001289726.1	For: CCCTTAAGAGGGATGCTGCC<br>Rev: TACGGCCAAATCCGTTCACA	124	Exons 1-2	1 µM	94
Actb	beta-actin	NM_007393.5	For: GCAAGCAGGAGTACGATGAGT<br>Rev: AGGGTGTAAAACGCAGCTCAG	88	Exon 6	1 µM	97

Supplementary Table 6. Antibodies used in protein expression analysis

Primary antibody			Secondary antibody		
Antigen	Brand (reference)	Dilution	Antigen IgG	Brand (reference)	Dilution
ATG5	Cell Signaling Technology (Cat# 12994)	1/1000	Anti-Rabbit IgG, HRP	Jackson ImmunoResearch (Cat# 711-035-152)	1/5000
ATG7	Cell Signaling Technology (Cat# 8558)	1/1000	Anti-Rabbit IgG, HRP	Jackson ImmunoResearch (Cat# 711-035-152)	1/5000
beclin-1	Cell Signaling Technology (Cat# 3495)	1/1000	Anti-Rabbit IgG, HRP	Jackson ImmunoResearch (Cat# 711-035-152)	1/5000
LC3B	Cell Signaling Technology (Cat# 2775)	1/1000	Anti-Rabbit IgG, HRP	Jackson ImmunoResearch (Cat# 711-035-152)	1/5000
SQSTM1/p62	Santa Cruz Biotechnology (Cat# sc-28359)	1/500	Anti-Mouse IgG, HRP	Jackson ImmunoResearch (Cat# 715-035-150)	1/5000
β-actin	Sigma-Aldrich (Cat# A5441)	1/5000	Anti-Mouse IgG, HRP	Jackson ImmunoResearch (Cat# 715-035-150)	1/5000

ATG, autophagy-related protein; LC3B, microtubule-associated protein 1 light chain 3; SQSTM1, sequestosome 1; HRP, horseradish peroxidase.

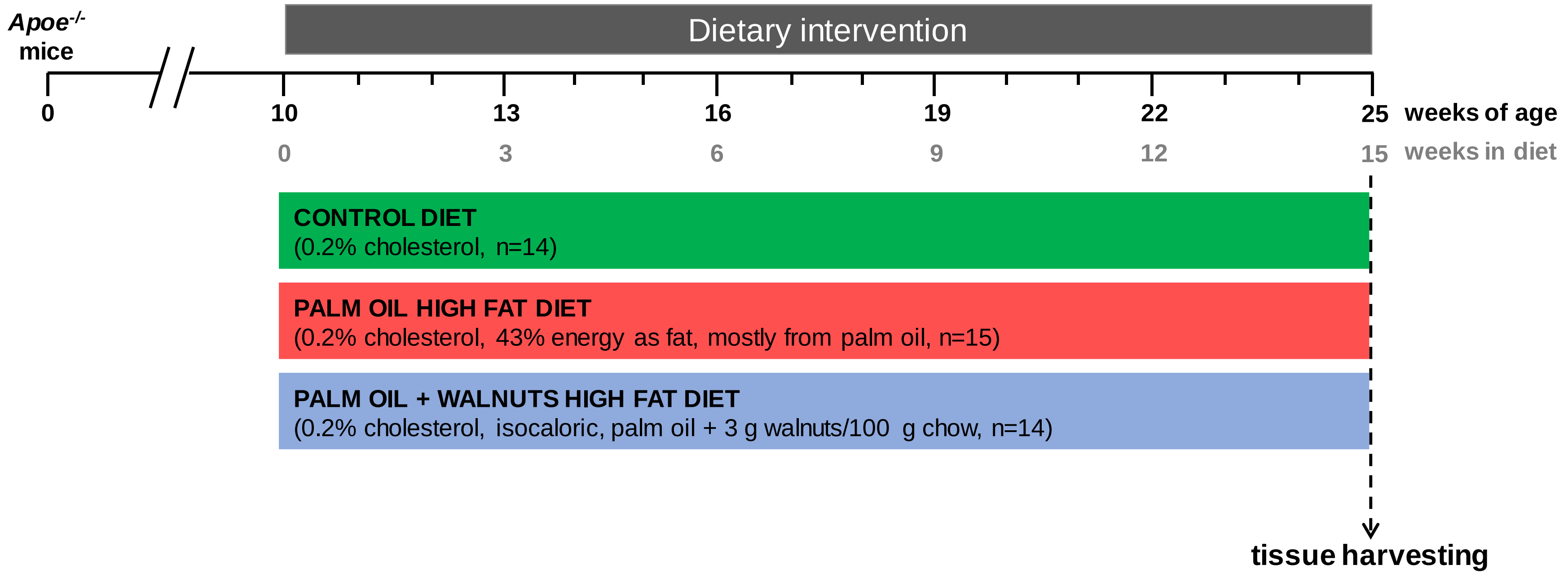

Supplementary Figure 1. Scheme of the experimental protocol of the dietary intervention in *Apoe*$^{-/-}$ male mice.

A

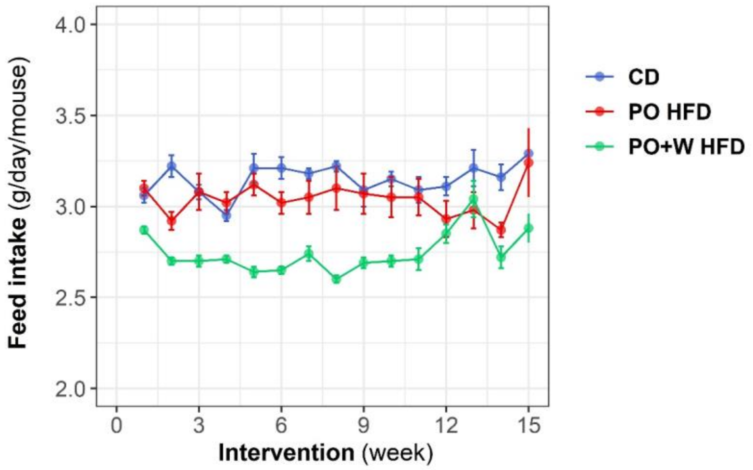

B

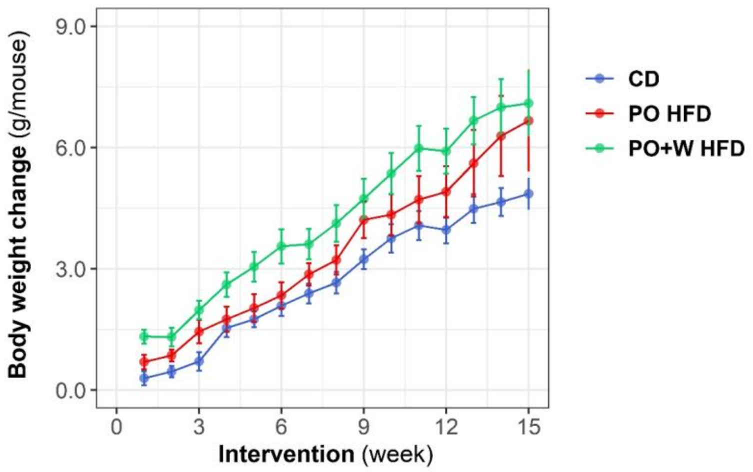

Supplementary Figure 2. Effects of dietary intervention on feed intake (A) and body weight changes (B) along the 15 weeks of dietary intervention in *Apoe*$^{-/-}$ mice. Line-plots show data as mean ± SEM. CD, control diet (n=14); PO HFD, palm oil-based high-fat diet (n=15; PO+W HFD, palm oil and walnuts high-fat diet (isocaloric inclusion of 3% of walnuts at expense of palm oil, n=14).

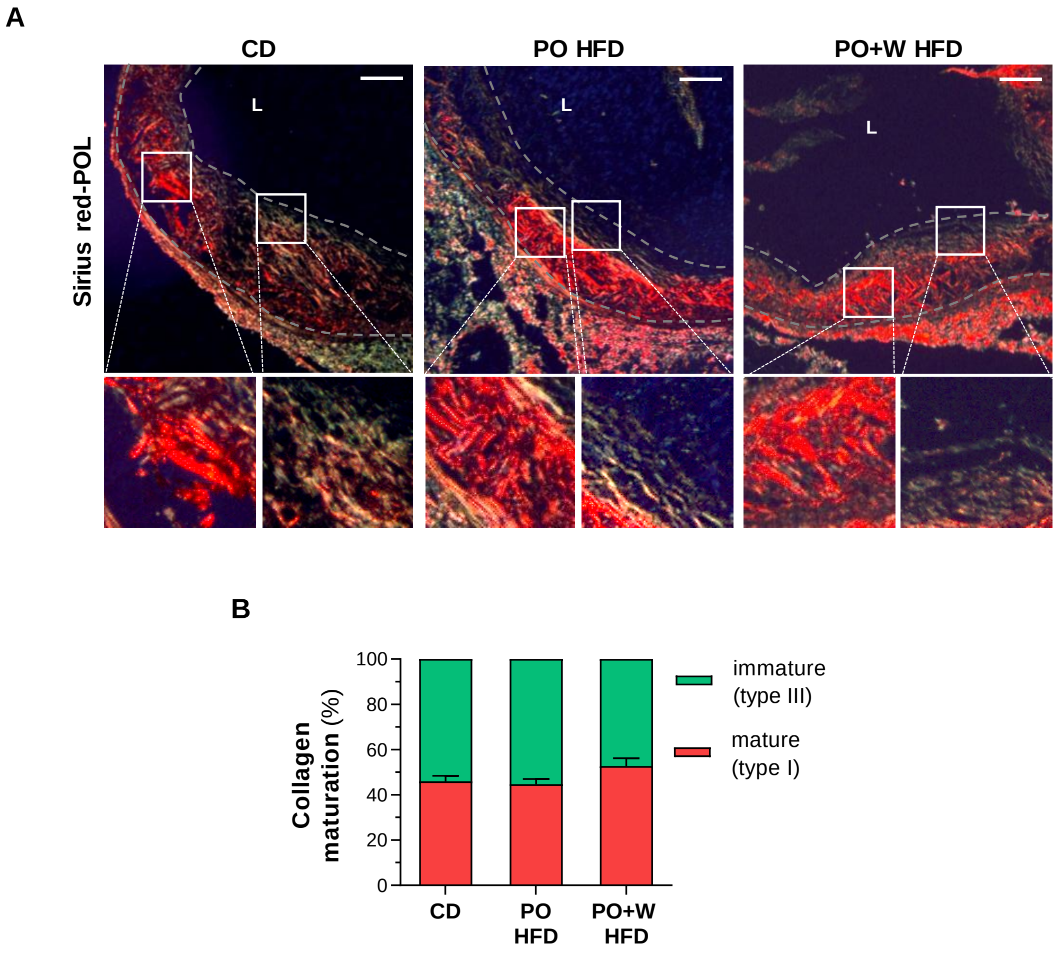

Supplementary Figure 3. Effect of dietary intervention on collagen maturation in atherosclerotic plaques from *Apoe*$^{-/-}$ mice. (**A**) Representative images (magnification x100; scale bars, 100 µm) of aortic root sections stained for collagen using Sirius red staining and visualized under polarised light (Sirius red-POL). High-magnification fields depict representative areas of mature (thicker orange-red fibers, type I) and immature (thinner yellow-green fibers, type III) collagen. (**B**) Quantitative analysis of collagen fibers composition as percentage of mature (type I) and immature (type III) collagen in atherosclerotic lesions. CD, control diet (n=14); PO HFD, palm oil-based high-fat diet (n=15); PO+W HFD, palm oil and walnuts high-fat diet (isocaloric inclusion of 3% of walnuts at expense of palm oil, n=14). Dashed white lines define atherosclerotic lesions. L, lumen.

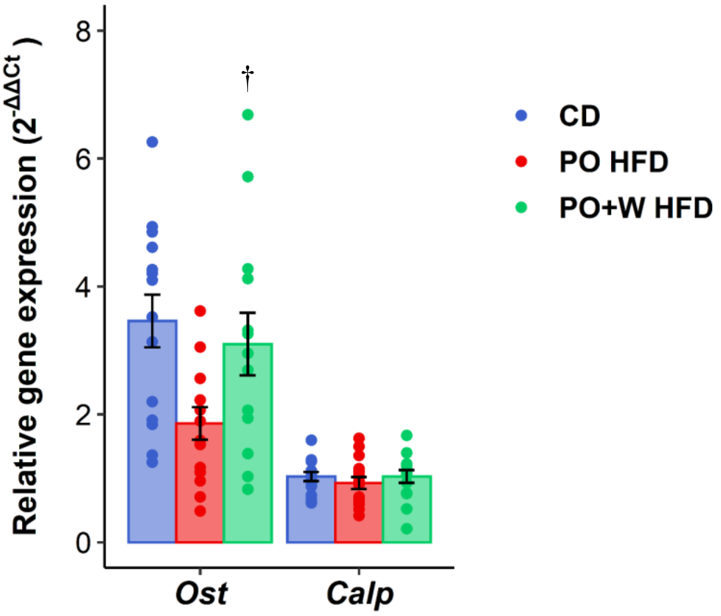

Supplementary Figure 4. Effect of dietary intervention on VSMC phenotypic switching in *Apoe*$^{-/-}$ mouse aorta. Quantitative real-time PCR analysis of synthetic (osteopontin, *Ost*) and contractile (calponin, *Calp*) VSMC phenotype markers in aortic tissue samples. Data normalized by GAPDH are expressed as relative gene expression (using $2^{-\Delta\Delta Ct}$ method). Bar plot show results as individual data points and mean ± SEM of the intervention groups. CD, control diet (n=14); PO HFD, palm oil-based high-fat diet (n=15); PO+W HFD, palm oil and walnuts high-fat diet (isocaloric inclusion of 3% of walnuts at expense of palm oil, n=14). *p*-values obtained by one-way ANOVA (after log-transformation) with Bonferroni post-hoc correction. †$p < 0.05$ vs. PO HFD.

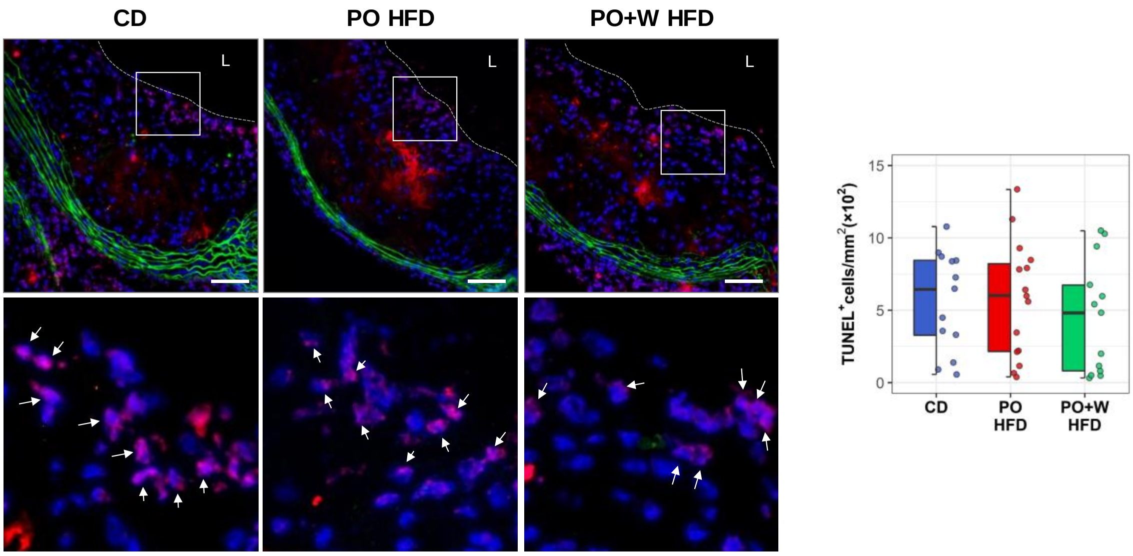

Supplementary Figure 5. Effect of dietary intervention on apoptosis in atherosclerotic lesions in *Apoe*$^{-/-}$ mice. Representative images (magnification x200; scale bars, 50 µm), high-magnification fields and quantitative analysis of apoptotic cells by using TUNEL assay in aortic root sections. Dashed lines define atherosclerotic lesions. Arrows indicate TUNEL-positive cells (purple). Dot and box plot shows individual values and depicts the median (horizontal bar), interquartile range (IQR, hinges) and 1.5×IQR (whiskers). CD, control diet (n=14); PO HFD, palm oil-based high-fat diet (n=15); PO+W HFD, palm oil and walnuts high-fat diet (isocaloric inclusion of 3% of walnuts at expense of palm oil, n=14). L, lumen.

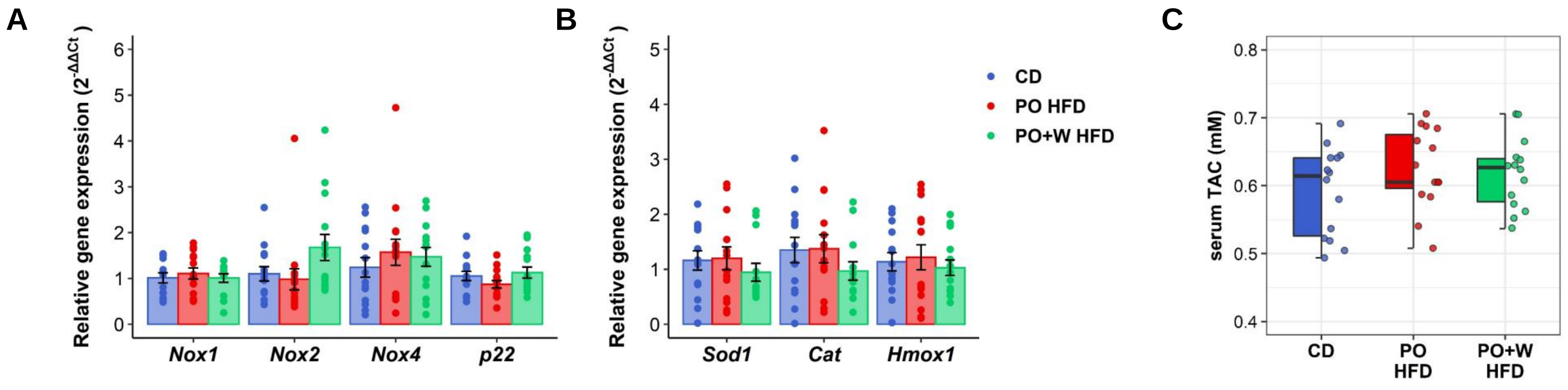

Supplementary Figure 6. Effect of dietary intervention on oxidative stress and antioxidant response gene expression in *Apoe*$^{-/-}$ mice. Quantitative real-time PCR analysis of Nox subunits (**A**) and antioxidant defense genes (**B**) in aortic tissue. Data normalized by GAPDH are expressed as relative gene expression (using $2^{-\Delta\Delta Ct}$ method). Results in bar plots are presented as individual data points and mean ± SEM of the intervention groups. (**C**) Measurement of total antioxidant capacity (TAC) in serum samples. Dot and box plot shows individual values and depicts the median (horizontal bar), interquartile range (IQR, hinges) and 1.5×IQR (whiskers). CD, control diet (n=14); PO HFD, palm oil-based high-fat diet (n=15); PO+W HFD, palm oil and walnuts high-fat diet (isocaloric inclusion of 3% of walnuts at expense of palm oil, n=14). $^{*}p<0.05$ compared with CD; $^{\dagger\dagger}p<0.01$ compared with PO HFD. *p*-values obtained by one-way ANOVA (after log-transformation) with Bonferroni post-hoc correction.

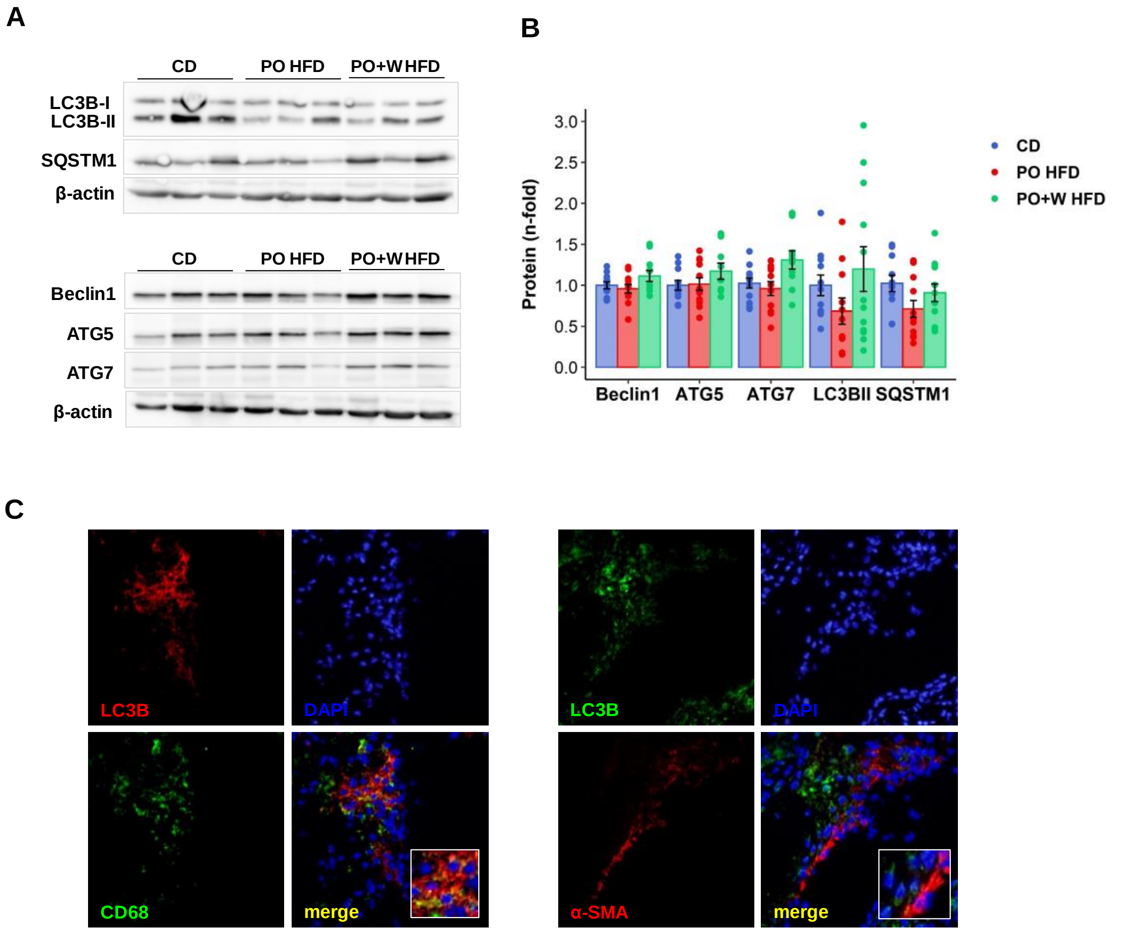

Supplementary Figure 7. Effect of dietary intervention on autophagy in *Apoe*$^{-/-}$ mice atherosclerotic lesions. Western blot analysis of autophagy-related proteins Beclin1, ATG5, ATG7, LC3B-II, SQSTM1 and β-actin (as loading control) in aortic protein extracts. Shown are representative images (**A**), and the summary of normalized quantification expressed as fold-increases *versus* control diet (**B**). Bar plot presents individual data points and mean ± SEM of the intervention groups. CD, control diet (n=12); PO HFD, palm oil-based high-fat diet (n=12); PO+W HFD, palm oil and walnuts high-fat diet (isocaloric inclusion of 3% of walnuts at expense of palm oil, n=12). (**C**) Representative images and high-magnification fields (rectangular areas) in atherosclerotic lesion sections showing immunodetection of CD68+ macrophages (left panel) and α-SMA+ VSMC (right panel), and colocalization with LC3B.

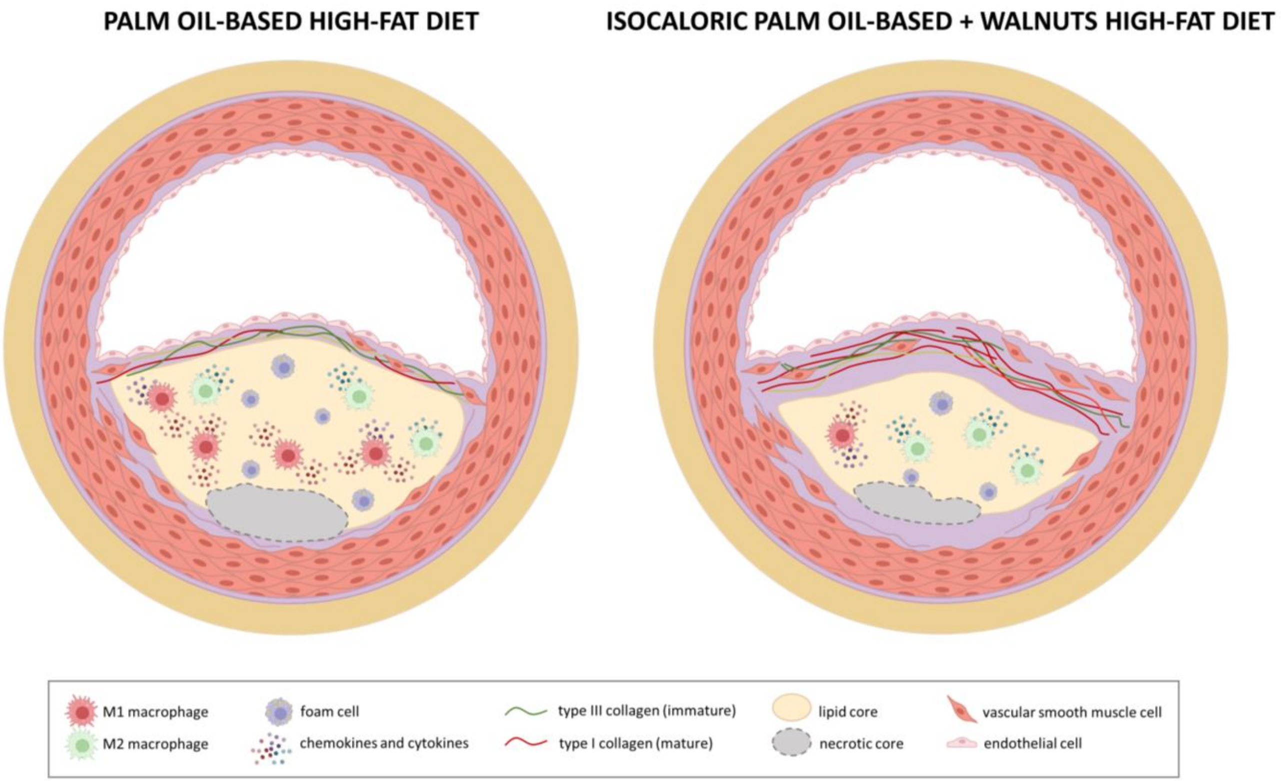

Supplementary Figure 8. In a background of unhealthy high-fat diet (based on palm oil, a source of saturated fat), *Apoe*-deficient mice developed a phenotype predicting unstable advanced atheroma plaques, characterized by a high lipid content and macrophage infiltration (with a higher proportion of pro-inflammatory M1 phenotype), low collagen content (mainly immature type III), necrotic areas, and a storm of inflammatory mediators (cytokines and chemokines) – LEFT. Partial replacement of palm oil with walnuts (without changing the total energy supplied as fat) did not reduce the size of the atherosclerotic lesion, but it resulted in a phenotype predicting more stable phenotype of atheroma plaque by impacting on lipid content, content and phenotype of macrophages, presence and type of collagen, and inflammatory response – RIGHT.
